# Contact between soft tick vectors of African swine fever virus and invasive wild pigs in the southeastern USA

**DOI:** 10.1186/s13071-025-06811-1

**Published:** 2025-05-12

**Authors:** Samantha M. Wisely, Carson Torhorst, Sebastian Botero-Cañola, Nicholas Canino, Angela M. James, Kathleen C. O’Hara

**Affiliations:** 1 Department of Wildlife Ecology and Conservation, University of Florida, Gainesville, FL USA; 2 United States Department of Agriculture, Animal and Plant Health Inspection Service, Veterinary Services, Fort Collins, CO USA

**Keywords:** Argasidae, Feral swine, Transboundary animal disease, Transmission dynamics, Vector-borne disease

## Abstract

**Background:**

African swine fever virus is a transboundary pathogen of high economic impact to the global pork industry. Florida has multiple factors that contribute to the high risk of introduction of African swine fever virus (ASFV) including high levels of commerce and human migration between Florida and Caribbean nations with ASFV, established backyard swine production, abundant populations of invasive wild pigs (*Sus scrofa*), and the presence of a soft tick species (*Ornithodoros turicata americanus*) that has been found to be a competent vector of ASFV in laboratory experiments. To better assess the hazard of ASFV vector-borne transmission in Florida, we documented contact between invasive wild pigs and *O. t. americanus* throughout Florida.

**Methods:**

We surveyed gopher tortoise (*Gopherus polyphemus*) burrows throughout Florida and collected *O. t. americanus* from infested burrows. To identify definitive contact between invasive wild pigs and soft ticks, we used established real time polymerase chain reaction primers and a probe to detect the deoxyribonucleic acid (DNA) of invasive wild pigs in the bloodmeals of *O. t. americanus.*. To detect potential wild pig–soft tick contact, we surveyed for evidence of pig activity within 5 m of an infested burrow entrance.

**Results:**

Across 61 sites, we found that 203 of 591 burrows (34%) were infested with the soft tick, *O. t. americanus*. Ten burrows across 57 sites (18%) had soft ticks with wild pig DNA in their abdomens. In total, 6 of 591 burrows (1%) had evidence of invasive wild pigs near the entrance. Three infested burrows had evidence of wild pigs near the entrance, one of these burrows also had soft ticks that were positive for wild pig DNA. Including both definitive and potential wild pig-soft tick contact, 12 of 61 sites (20%) had evidence of wild pig–soft tick association.

**Conclusions:**

In Florida, contact between invasive wild pigs, a potential reservoir for ASFV, and *O. t. americanus*, a competent vector, was measurable and occurred throughout the distribution of the vector. Florida is at risk not only for ASFV emergence but establishment of this pathogen in a sylvatic cycle. In addition to managing invasive wild pigs, future ASFV response planning needs to include plans for surveying and managing vector populations should an outbreak occur.

**Graphical Abstract:**

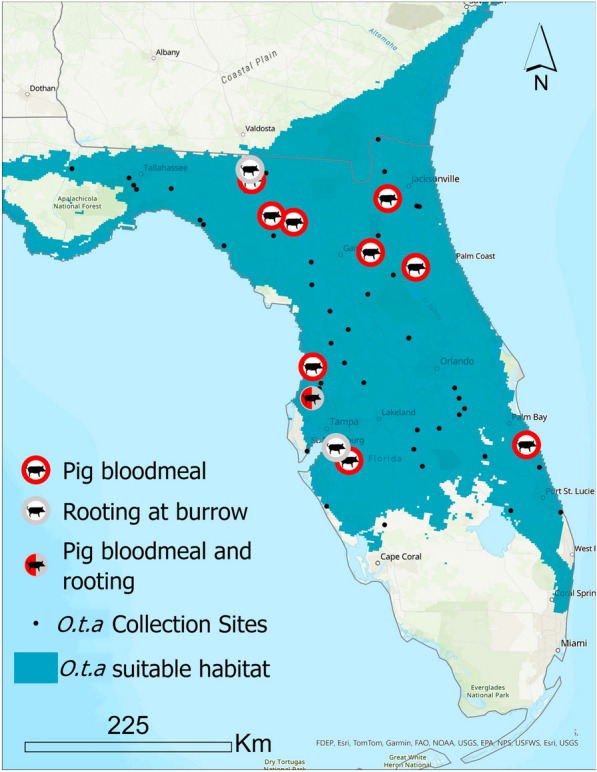

**Supplementary Information:**

The online version contains supplementary material available at 10.1186/s13071-025-06811-1.

## Background

African swine fever (ASF) is an infectious viral hemorrhagic disease of domestic and wild pigs (*Sus scrofa*) that has spread globally and has profoundly impacted the pork industry throughout southeast Asia and large parts of Europe [1]. While the vast majority of transmission events of African swine fever virus (ASFV) occur directly between live animals or indirectly from infected pork products or fomites, vector-borne transmission from *Ornithodoros* ticks also occurs [2]. In Africa, where ASFV is endemic, *Ornithodoros* ticks are a part of the sylvatic cycle in multiple species of wild suids [3]. In Europe, *Ornithodoros* ticks have been implicated in local re-emergence of the virus [4]. ASFV was found in *Ornithodoros erraticus* (Acari: Argasidae) 5 years after infected pigs were removed from this region of Portugal and ASFV infected ticks were found at 3 of 34 farms investigated, suggesting that infected ticks could maintain a sylvatic cycle of ASFV transmission [5].

Although not in the USA, ASFV re-emerged on the island of Hispañola in the Caribbean basin in 2021 after a 40-year absence [1]. Given the large exchange of people and goods between Florida and Caribbean islands, Florida is considered a high risk for ASFV introduction via the importation of infected pork products or contaminated fomites [1]. Exacerbating the risk of an outbreak in Florida are the nearly 1,000,000 invasive wild pigs (*Sus scrofa*) that live throughout the state [6]. This vertebrate pest is a habitat and diet generalist that is ubiquitous to the upland forests and bottomlands of the southeastern Gulf Forest ecosystem [7]. Should wild pigs in Florida become infected and an outbreak ensue, eradication of ASFV would become more complicated than just eliminating the virus from domestic operations [8]. This scenario has already played out in Europe, where native European boar maintain a sylvatic cycle of the pathogen that spills back and forth between domestic pigs and European boar [9]. Adding to this complicated disease system is the poorly understood role that an endemic soft tick species might play in transmission dynamics, should the virus emerge in Florida [8].

At least three species of *Ornithodoros* ticks in the USA, including *O. turicata* collected from Florida, have been found to be competent vectors of ASFV in experimental laboratory conditions [10]. *Ornithodoros turcata* is considered a species of high risk for becoming involved in ASFV transmission should it emerge in the USA because of its widespread distribution and propensity for feeding on numerous vertebrate species [3]. Previous studies from Texas have shown that *O. turicata* lives in a variety of microhabitats including animal burrows, crevices, and caves, and feeds occasionally on invasive wild pigs [11, 12]. Preliminary investigations indicate, however, that *O. turicata americanus*, an eastern subspecies found predominantly in Florida, has different ecological and biological characteristics [13]. In Florida, these nidicolous ticks are found almost exclusively in gopher tortoise (*Gopherus polyphemus*) burrows [14–16] which occur predominantly in upland forests in the Gulf Forest ecosystem [17]. Thus, there is overlap in distribution and habitat use of the arthropod vector of ASFV and its vertebrate host, *Sus scrofa* in Florida. This overlap, however, does not imply contact; *O. t. americanus* would need to feed on invasive wild pigs to create a risk for a sylvatic transmission cycle. To better assess the risk of ASFV vector-borne transmission in Florida, a region at high-risk for the importation of ASFV, we documented contact between invasive wild pigs and the ASFV-competent vector, *O. t. americanus*, throughout the state. These observations provide an indicator of the amount of host–vector association.

## Methods

**Fig. 1:**
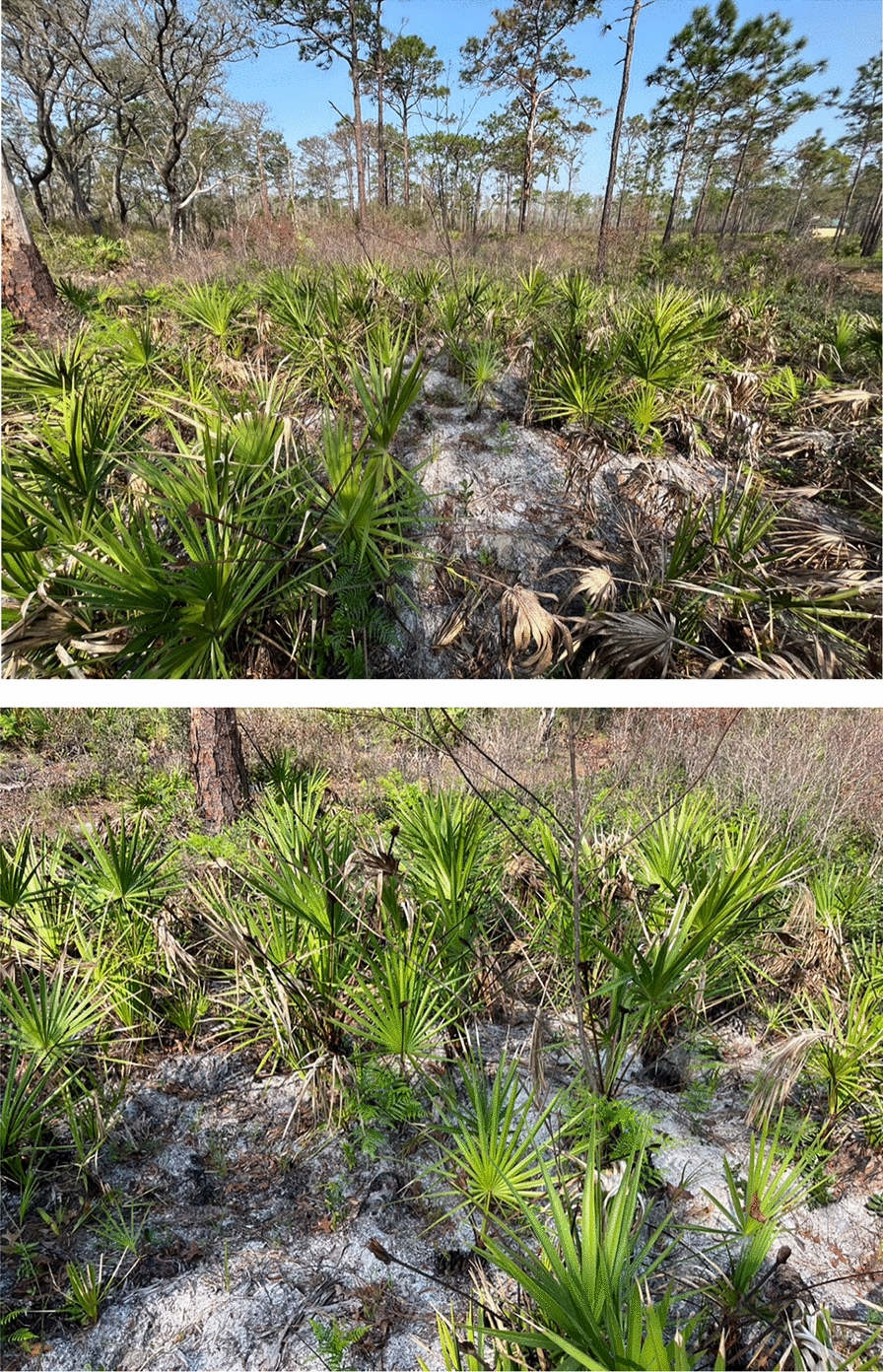
Photographs of rooting by invasive wild pigs at a gopher tortoise (*Gopherus polyphemus*) burrow in the flatwood forest ecosystem of Florida. The top photo shows the burrow and rooting in the context of the ecosystem. The bottom photo contrasts the rooted ground with overturned sandy soil in the foreground with the leaf litter-covered hardpacked soil of the undisturbed ground in the background. Photo courtesy of C.T.

We used two methods for determining wild pig–soft tick contact. We surveyed for definitive evidence of wild pig–tick contact by determining if soft ticks had ingested pig blood using a pig-specific, real time polymerase chain reaction (rtPCR) molecular assay. We surveyed for potential wild pig-soft tick contact by determining if gopher tortoise burrows, a known microhabitat of *O. t. americanus*, had wild pig activity within 5 m of the burrow entrance. We chose 5 m because that was within the distances (4–8 m) at which a CO_2_ source attracted *O. t. americanus* out of a gopher tortoise burrow [18]. Rooting by invasive wild pigs was highly visible and recognizable in the sandy soil of upland Florida habitats where gopher tortoise burrows were located (Fig. 1).

As part of a systematic survey of the distribution of *O. t. americanus* in Florida [15], we surveyed 591 gopher tortoise burrows at 113 sites. Each site was 10 km × 10 km and was chosen because it had at least one known gopher tortoise burrow location and was located on public property. Sites were stratified across the major ecoregions of Florida. We chose to survey at gopher tortoise burrows because soft ticks are nidicolous and remain in host nests and burrows, and *O. t. americanus* has previously been associated with the burrows of gopher tortoises [19]. At each burrow we noted if there was pig rooting within 5 m of the burrow entrance. Rooting behavior is a foraging tactic for arthropod larvae, fossorial mammals, reptiles, and amphibians, as well as vegetative forage, and the behavior mobilizes soil up to a meter deep and is highly visible (Fig. 1, [20]).

We collected soft ticks from unoccupied burrows with a modified leaf vacuum (Homelite 26 cc gas-powered blower/vacuum, 4.25 to 11.3 m^3^/min) designed to collect substrate from all sides of the first meter inside of burrows. For each burrow we filled a 3.8-L resealable polyethylene bag (Ziploc, S.C. Johnson & Son, Racine, WI) with substrate that weighed 1–3 kg. In the lab we used a series of sieves to separate soft ticks from the substrate. Full details of the collection method including a comparison of the probability of detecting soft ticks by various collection methods and environmental conditions are published [16]. Soft ticks were morphologically identified to species level using entomological keys [21], and nymphs and adults were stored in 95% molecular grade ethanol at −20 °C until DNA extraction.

To identify the bloodmeals of soft ticks, we pooled ticks by burrow in pools of a maximum of 5 and analyzed a maximum of 50 ticks per burrow (ten pools). We extracted DNA from tick pools using the Gentra Puregene DNA extraction kit (Qiagen, Hilden, German) in a lab dedicated to working with low yield, low molecular weight DNA. Prior to DNA extraction, ticks were washed using a solution of 10% bleach. Each tick was submerged in the bleach solution for 15 s. Ticks were then submerged for 15 s in two separate deionized water washes to remove any remaining bleach or environmental residue from their exoskeleton. Ticks were then bisected using a sterile scalpel blade before being placed in their respective pools in 1.5 mL microcentrifuge tubes (Thermo Fisher Scientific, Waltham, MA, USA) with 600 µL of Puregene Cell Lysis Solution (Qiagen, Hilden, German). We modified the manufacturer’s recommended protein digestion protocol [22] by incubating the pooled ticks for 24 h in cell lysis buffer at room temperature. Proteinase K (20uL, Millipore Sigma, Darmstadt, Germany) was then added to each pool of ticks and incubated for 24 h at room temperature. An additional 20uL of proteinase K was added to the pooled ticks and cell lysis solution and incubated at 56 ℃ to complete the protein digestion. DNA extraction was then completed following the manufacturer’s protocol.

We amplified and quantified a 176 base pair (bp) segment of the *cytb* gene using previously published primers and a probe and real time PCR (rtPCR) conditions that were determined to be species-specific to wild boar (*Sus scrofa)* mitochondrial DNA (mtDNA) [23]. We included a positive control of extracted DNA from an invasive wild pig collected in Florida and a negative control of molecular-grade water in each 96-well plate of assays. To further assess the specificity of the assay to the local vertebrate community, we included the DNA of the following 13 vertebrates in one set of assays: Virginia opossum (*Didelphis virginiana*), raccoon (*Procyon lotor*), nine-banded armadillo (*Dasypus novemcinctus*), coyote (*Canis latrans*), eastern gray squirrel (*Sciurus carolinensis*), cotton mouse (*Peromyscus gossypinus*), eastern woodrat (*Neotoma floridana*), Florida mouse (*Podomys floridanus*), golden mouse (*Ochrotomys nuttalli*), eastern harvest mouse (*Reithrodontomys humulis*), hispid cotton rat (*Sigmodon hispidis*), house mouse (*Mus musculus*), and Norway rat (*Rattus norvegicus*).

## Results

Of the 591 burrows that were surveyed for soft ticks, 203 burrows were infested with at least one soft tick. A total of 3066 soft ticks that were morphologically identified as *O. t. americanus* were collected from substrate at the surface to 1 m deep in the burrow. The average soft tick abundance per burrow was 15 ± 2.3 soft ticks with a maximum of 232 ticks found in one substrate sample.

**Fig. 2:**
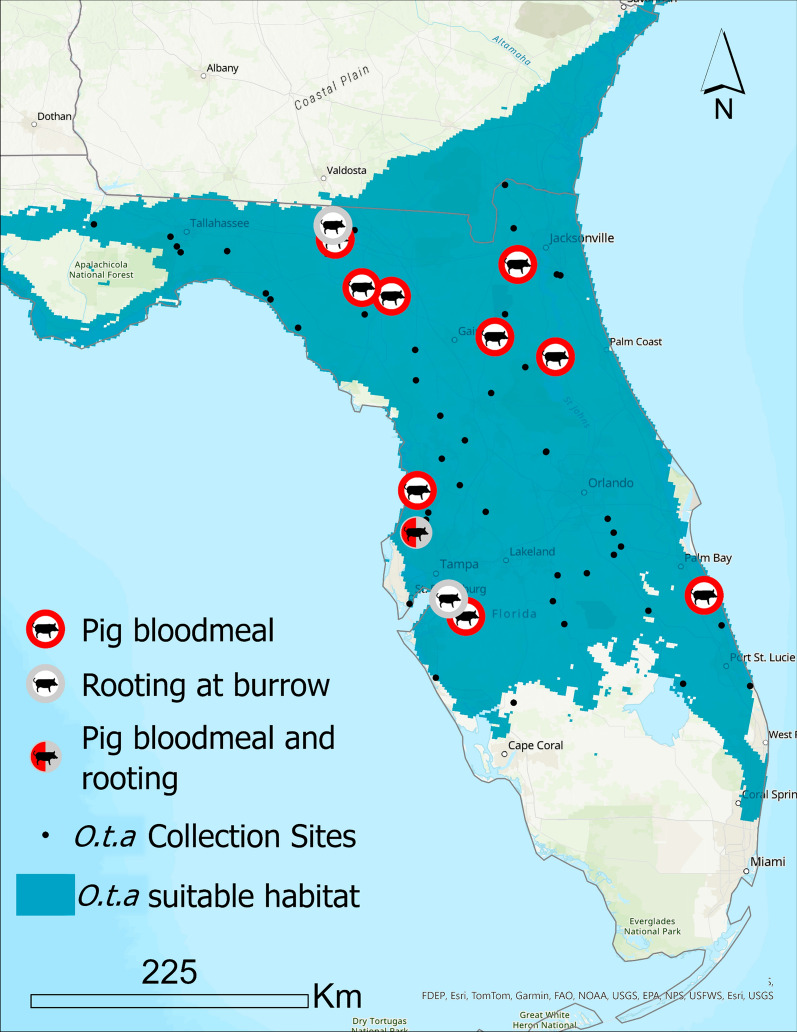
Map of pig–soft tick associations. The soft tick, *Ornithodoros turicata americanus* (*O.t.a.* in the legend) is broadly distributed (green shading) throughout Florida [10]. We found ten gopher tortoise (*Gopherus polyphemus*) burrows that were infested with soft ticks that had taken a wild pig bloodmeal (red circle). In addition, four soft tick infested burrows had indications that wild pigs were rooting near the burrow entrances. Of the 61 infested burrows, we found 12 (20%) that had pig–soft tick associations

Using rtPCR, we tested 2978 soft ticks in 744 pools from 193 of the 203 infested burrows (95%) to determine if they had evidence of an ingested bloodmeal from a wild pig. Twelve soft tick pools from 11 of the 193 burrows (6%) were positive for wild pig blood as detected by rtPCR. One burrow had two pools of soft ticks that were positive for wild pig blood. The 203 infested burrows were located at 61 sites throughout Florida (Fig. 2). Ticks in burrows were tested at 57 of the 61 sites (93%). Overall, 10 of the 57 sites (18%) had one burrow with soft ticks that fed on pigs; 1 site had two burrows with wild pig bloodmeals. The rtPCR primers and probe did not amplify any DNA except from that of *Sus scrofa*.

Each of the 591 burrows was also surveyed for signs of pig disturbance (rooting) within 5 m of the burrow entrance. In total, 6 of the 591 burrows (1%) had evidence of pig rooting, 3 of 203 infested burrows (1%) had evidence of pig rooting at the entrance, and 1 of the infested burrows (< 1%) also had 2 pools of ticks that were positive for pig–tick contact by rtPCR. Including the number of sites with both definitive contact (evidence of wild pig–tick contact by rtPCR) or potential contact (wild pig rooting near an infested burrow entrance), a total of 12 of 61 sites (20%) had evidence of wild pig–soft tick association.

## Discussion

The objectives of this study were to determine if contact occurs between the host and vector of ASFV, and to map the locations where this contact occurred. This study found that throughout its distribution in Florida the soft tick, *O. t. americanus*, fed on wild pigs (Fig. 2), and this finding establishes an additional potential route of infection for ASFV in wild pigs in Florida. Of 61 sites surveyed for wild pig–soft tick contact (including both definitive and potential contact), we found evidence of contact at 20% of the sites (12 sites).

Transmission requires contact between host and vector [24]. Understanding that contact between host and vector occurs over a large spatial area provides a better understanding of the hazard that soft ticks present in ASFV epidemiology and risk of transmission. To that end, recent surveys in Florida found that the vector, *O. t. americanus*, is broadly distributed across 62% of the land mass of Florida [15, 16]. Coupled with the ubiquitous distribution of invasive wild pigs [6], we hypothesized that contact between these hosts and vectors of ASFV would occur. Indeed, we found that 20% of the sites we surveyed throughout Florida had evidence of wild pig–soft tick contact, which provides the baseline for future entomological studies.

This study had several limitations. The nidicolous lifestyle and short feeding time of soft ticks has made studies of this taxon’s diversity, ecology, and distribution challenging, and an understanding of its role as a vector for both human and animal pathogens elusive [8]. Surveillance was limited to gopher tortoise burrows which may not represent the breadth of microhabitats available to soft ticks, such as pig styes or other microhabitats near domestic pig facilities, and thus may underestimate the risk for pig–soft tick contact. In addition, the assay we used cannot distinguish between wild or domestic pigs [23]. All the locations that we surveyed were located on public lands and not near domestic pig farms, and thus we presumed the bloodmeals to be from invasive wild pigs which are defined as both feral swine and non-native Eurasian boar [25].

## Conclusions

Florida is at high risk for the emergence of ASFV [1]. The large population size of invasive wild pigs, the vast distribution of competent vectors for ASFV, and the measurable contact that occurs between wild pigs and soft ticks suggest that Florida is at risk not only for ASFV emergence but for establishment of this pathogen in a sylvatic cycle if ASFV were introduced into the USA. Future ASFV response planning should consider including plans for surveying and managing both host and vector populations should an outbreak occur.

## Supplementary Information

Additional file 1. Dataset S1. Occurrence of soft ticks in burrows and occurrence of pig DNA in tick pools. Supplementary_data_OTA.xls

## Data Availability

All data for this manuscript are available in the Supplementary Information.

## Abbreviations

ASF: African swine fever
ASFV: African swine fever virus
DNA: Deoxyribonucleic acid
mtDNA: Mitochondrial deoxyribonucleic acid
rtPCR: Real time polymerase chain reaction
